# Energy and economic dataset of the worldwide optimal photovoltaic-wind hybrid renewable energy systems

**DOI:** 10.1016/j.dib.2020.106476

**Published:** 2020-11-01

**Authors:** Domenico Mazzeo, Cristina Baglivo, Nicoletta Matera, Pierangelo De Luca, Paolo Maria Congedo, Giuseppe Oliveti

**Affiliations:** a Department of Mechanical, Energy and Management Engineering (DIMEG), University of Calabria, P. Bucci Cube 46/C, It, 87036 Arcavacata of Rende, CS, Italy; b Department of Engineering for Innovation, University of Salento, Via per Arnesano, It, 73100 Lecce, Italy

**Keywords:** Cost-optimal, Grid-connected, Hybrid renewable systems, Optimization, PV-wind hybrid system, Stand-alone, TRNSYS, Worldwide mapping

## Abstract

The data describe supplementary materials supporting the research article entitled “Worldwide geographical mapping and optimization of stand-alone and grid-connected hybrid renewable system techno-economic performance across Köppen-Geiger climates” (Mazzeo et al., 2020). Hybrid renewable energy systems are increasingly adopted worldwide as technically and economically effective solutions to achieve energy decarbonization and greenhouse gas reduction targets. This data article includes the results of worldwide techno-economic optimization of stand-alone and grid-connected photovoltaic-wind hybrid renewable energy systems designed to meet the electrical energy needs of an office district. The technical simulations have been performed in TRNSYS 17 (Transient Energy System) environment. A total of 48 different locations around the world have been chosen across Köppen-Geiger climates with different latitudes and homogeneously distributed over the whole globe, considering very different climates. The analyses have been conducted for 343 different system power configurations, considering both stand-alone and grid-connected systems. A total of 16464 dynamic simulations were performed, summarized in yearly energy output from each component and in energy and economic indicators.

## Specifications Table

**Table:** 

Subject	Renewable Energy, Environmental Sustainability
Specific subject area	Hybrid system design
Type of data	Tables, graphs and maps
How data were acquired	- Technical data: Parametric simulations and optimization - Economic data: Parametric simulations and optimization
Data format	Raw
Parameters for data collection	For all locations, the energy and economic data were obtained by using the following parameters: • Climatic data: air temperature, horizontal solar radiation and wind speed; • Technical data: electrical and thermal photovoltaic parameters, wind and battery electrical parameters in the reference conditions; • Economic data: cost of the renewable system, electricity cost and solar and wind feed-in-tariff price.
Description of data collection	1. All analyses have been conducted for 48 locations characterized by different latitudes and homogeneously distributed around the world. The typical meteorological year was used to extract the environmental variables. 2. Technical parameters and costs of each HRES (hybrid renewable energy system) component have been selected. Worldwide electricity prices and feed-in-tariff subsidies have been collected. 3. Four datasets have been developed, containing techno-economic parameters and indicators related to: - worldwide energetically optimal SA (stand-alone) PV-wind HRES; - worldwide economically optimal SA PV-wind HRES; - worldwide energetically optimal GC (grid-connected) PV-wind HRES; - worldwide economically optimal GC PV-wind HRES. 4 For each location and dataset, the optimal HRES power configuration has been found among 343 ones obtained by changing the PV and wind power installed and by varying the storage capacity of the battery system. The data provided originate from 16464 simulations carried out in TRNSYS software of 343 HRES power configurations in 48 different locations. The energy optimization has been performed to find, in each locality, the system power configuration that guarantees a high percentage of satisfied load and the maximum utilization of the energy produced for the SA HRES and the minimum level of energy exchange with the grid for the GC HRES. Furthermore, two economic optimization problems were formulated, respectively, for the SA and GC HRES, to select the system power configuration with the highest benefit-cost ratio.
Data source location	Around the world across Köppen-Geiger climates
Data accessibility	With the article
Related research article	D. Mazzeo, N. Matera, P. De Luca, C. Baglivo, P. M. Congedo, G. Oliveti, Worldwide mapping and optimization of stand-alone and grid-connected hybrid renewable system performance across Köppen‐Geiger climates, Applied Energy, 2020, https://doi.org/10.1016/j.apenergy.2020.115507[1].

## Value of the Data

• These data are useful for identifying the optimal energy and economic configurations for each location around the world, considering both SA and GC systems. In addition, they provide the best localities in the world to host SA and GC HRES. Finally, data constitute a concrete device and database to detect the techno-economic performance of the optimal hybrid system around the world.
• Maps are useful for rapid evaluation and comparison of the distribution of the techno-economic performance of the optimal HRES from a geographical point of view.
• Data can be used by other researchers as a reference to compare investigations in any location worldwide, by stakeholders, entrepreneurs and policymakers.
• The database can be extended to further localities worldwide, can be used to design installations of a hybrid system and to draw up economic incentive plans.
• Dataset represents a device to inform people, researchers, stakeholder, entrepreneurs and policymaker where it is reliable and profitable to install an HRES.
• In remote or isolated locations or underdeveloped countries, without access to the electricity, data can help to spread reliable and profitable SA installations. In urban areas with a grid connection, data are a valid guideline to reduce energy interaction between the user and grid, and consequently to avoid a grid overload, and to create new profitable investments for a private owner or public users.

## 1 Data Description

Table 1 lists all 48 localities chosen across Köppen-Geiger climates [2,3]. The localities have different latitudes and are homogeneously distributed around the world, as follows:

- eight locations belong to climate group A (tropical);
- six to group B (dry);
- eighteen to group C (temperate);
- sixteen to group D (continental).

**Table 1:** Localities chosen across Köppen-Geiger climates.

Locality	Country	Koppen classification
Toamasina	Madagascar	Af
Singapore	Singapore	Af
Recife, Pernambuco	Brazil	Am
Miami, Florida	United States	Am
Lihue, Hawaii	United States	As
Mombasa	Kenya	As
Caracas	Venezuela	Aw
Kano	Nigeria	Aw
Baghdad	Iraq	BWh
Cairo	Egypt	BWh
Kabul	Afghanistan	BSk
Baku	Azerbaijan	BSk
Odessa, Texas	United States	BSh
Maracaibo	Venezuela	BSh
Buenos Aires	Argentina	Cfa
Milan	Italy	Cfa
Berlin	Germany	Cfb
London	United Kingdom	Cfb
Vancouver, British Columbia	Canada	Cfb
Melbourne, Victoria	Australia	Cfb
Bogotá, Cundinamarca	Colombia	Cfb
Wellington	New Zealand	Cfb
Reykjavík	Iceland	Cfc
Auckland Islands	New Zealand	Cfc
Rome	Italy	Csa
Adelaide	Australia	Csa
Porto	Portugal	Csb
La Coruna	Spain	Csb
New Delhi	India	Cwa
Hong Kong	China	Cwa
Johannesburg	South Africa	Cwb
Nairobi	Kenya	Cwb
Bucharest	Romania	Dfa
Toronto, Ontario	Canada	Dfa
Moskva	Russia	Dfb
Ottawa, Ontario	Canada	Dfb
Tromsø	Norway	Dfc
Anchorage,Alaska	United States	Dfc
Oymyakon, Sakha Republic	Russia	Dfd
Verhojansk, Sakha Republic	Russia	Dfd
Hakkâri	Turkey	Dsa
Cambridge Bay, Nunavut	United States	Dsa
Dras	India	Dsb
Flagstaff, Arizona	United States	Dsb
Beijing	China	Dwa
Seoul	South Korea	Dwa
Pyongyang	North Korea	Dwb
Vladivostok	Russia	Dwb

The typical meteorological year file .tm2, available in the TRNSYS library, is used for all simulations.

The datasheets reported in the file “**01 Supplementary material**” contain techno-economic parameters and indicators related to:

- worldwide energetically optimal SA PV-wind HRES;
- worldwide economically optimal SA PV-wind HRES;
- worldwide energetically optimal GC PV-wind HRES;
- worldwide economically optimal GC PV-wind HRES.

For all locations, the technical, energy and economic data are reported in terms of:

- Nominal PV, wind and battery power;
- PV, wind, battery, load and overall power fraction;
- Yearly energy produced by the PV and wind generator;
- PV and wind energy fraction;
- Yearly energy: sent directly to the load, drawn from the battery, missing, drawn from the grid, dissipated, produced sent to the grid;
- PV, wind, inverter and battery capital costs;
- Overall system cost;
- Cost saving;
- PV and wind revenue;
- Yearly benefit;
- Satisfied load fraction;
- Utilization factor;
- Grid Energy Interaction Factor;
- Benefit cost ratio.

All parameters or indicators are explained in detail in the nomenclature sheet.

The “**02 Supplementary material – Histograms**” represents a graphical elaboration of some parameters or indicators reported in the 01 Supplementary material”. It contains the following figures:

• Fig. S.1. PV and wind power fractions for the energetically optimal stand-alone and grid-connected systems.
• Fig. S.2. PV and wind power fractions for the economically optimal stand-alone and grid-connected systems.
• Fig. S.3. Battery power fractions and load power fractions for the energetically optimal stand-alone and grid-connected systems.
• Fig. S.4. Battery power fractions and load power fractions for the economically optimal stand-alone and grid-connected systems.
• Fig. S.5. Wind and PV energy generated by the energetically optimal stand-alone and grid-connected systems.
• Fig. S.6. Wind and PV energy generated by the economically optimal stand-alone and grid-connected systems.
• Fig. S.7. PV and wind fractions of the overall energy generated in the energetically optimal stand-alone and grid-connected systems.
• Fig. S.8. PV and wind fractions of the overall energy generated in the economically optimal stand-alone and grid-connected systems.
• Fig. S.9. PV and wind fractions of the overall energy generated in order of yearly average wind speed strength (decreasing from left to right) for the energetically optimal stand-alone and grid-connected systems.
• Fig. S1.10. PV and wind fractions of the overall energy generated in order of yearly average horizontal solar radiation strength (decreasing from left to right) for the energetically optimal stand-alone and grid-connected systems.
• Fig. S1.11. PV and wind fractions of the overall energy generated in order of yearly average wind speed strength (decreasing from left to right) for the economically optimal stand-alone and grid-connected systems.
• Fig. S1.12. PV and wind fractions of the overall energy generated in order of yearly average horizontal solar radiation strength (decreasing from left to right) for the economically optimal stand-alone and grid-connected systems.

The following Fig. 1a, Fig. 1b, Fig. 2a, Fig. 2b, Fig. 3a, Fig. 3b, Fig. 4a, Fig. 4b, Fig. 5a, Fig. 5b, Fig. 6a, Fig. 6b, Fig. 7a, Fig. 7b, Fig. 8a, Fig. 8b, Fig. 9a, Fig. 9b show further worldwide geographical maps of the energy performance and economic profitability of the energetically and economically optimal HRES, resulting from the analysis of the data presented in the “01 Supplementary material”. In general, they show the worldwide geographical mapping of the:

• PV/wind energy generated by the energetically/economically optimal SA and GC systems;
• overall energy generated by the energetically/economically optimal SA and GC systems;
• energy generated by the energetically/economically optimal SA and GC systems sent directly to the load.
• energy drawn from the battery in the energetically/economically optimal SA and GC systems;
• energy missing in the energetically/economically optimal SA systems and drawn from the grid in the energetically/economically optimal GC systems.
• energy in excess dissipated by the energetically/economically optimal SA systems and sent to the grid by the energetically/economically optimal GC systems.
• cost of the energetically/economically optimal SA and GC systems.
• benefit from the energetically/economically optimal SA and GC systems.

**Fig. 1a:**
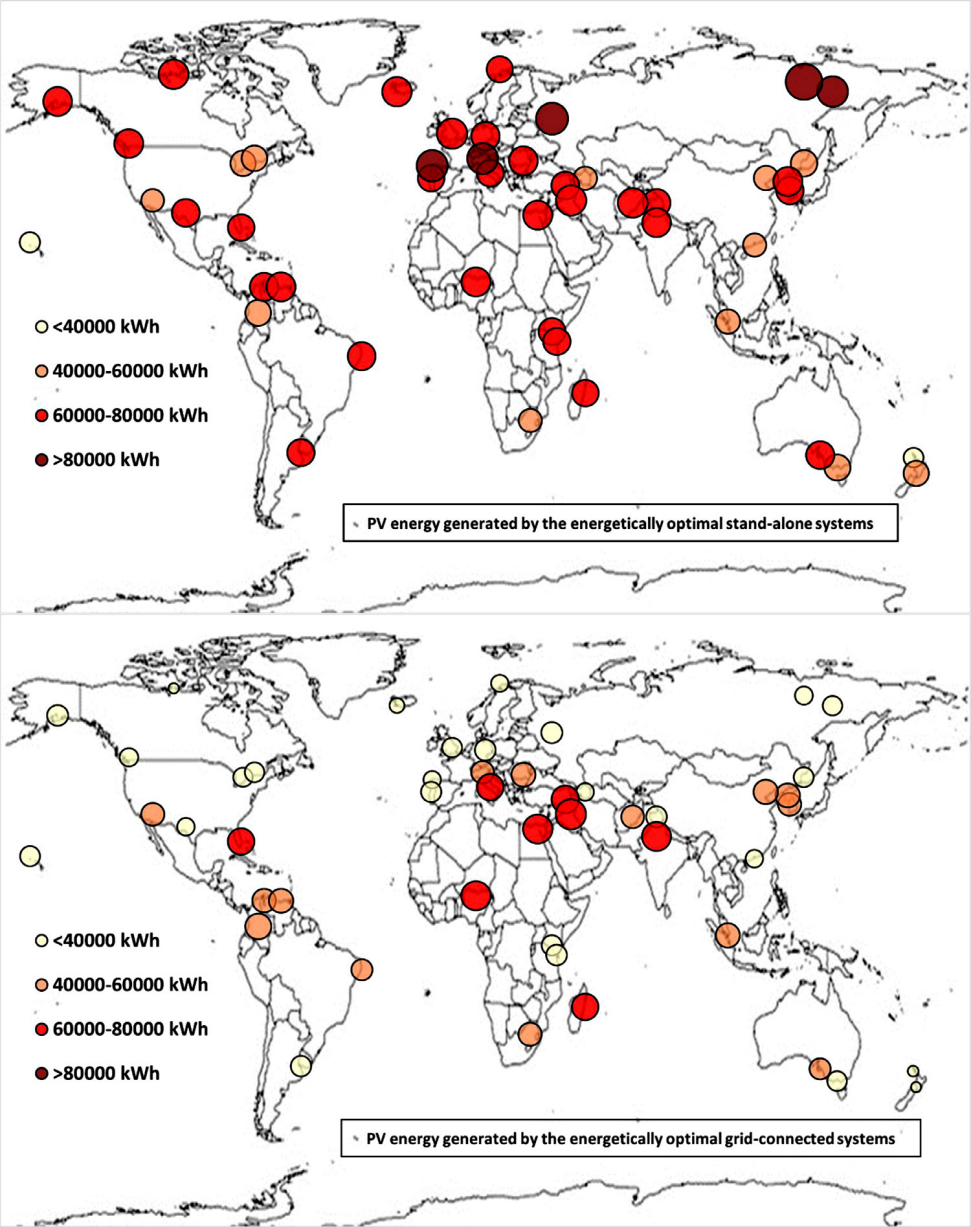
Worldwide mapping of the PV energy generated by the energetically optimal stand-alone and grid-connected systems.

**Fig. 1b:**
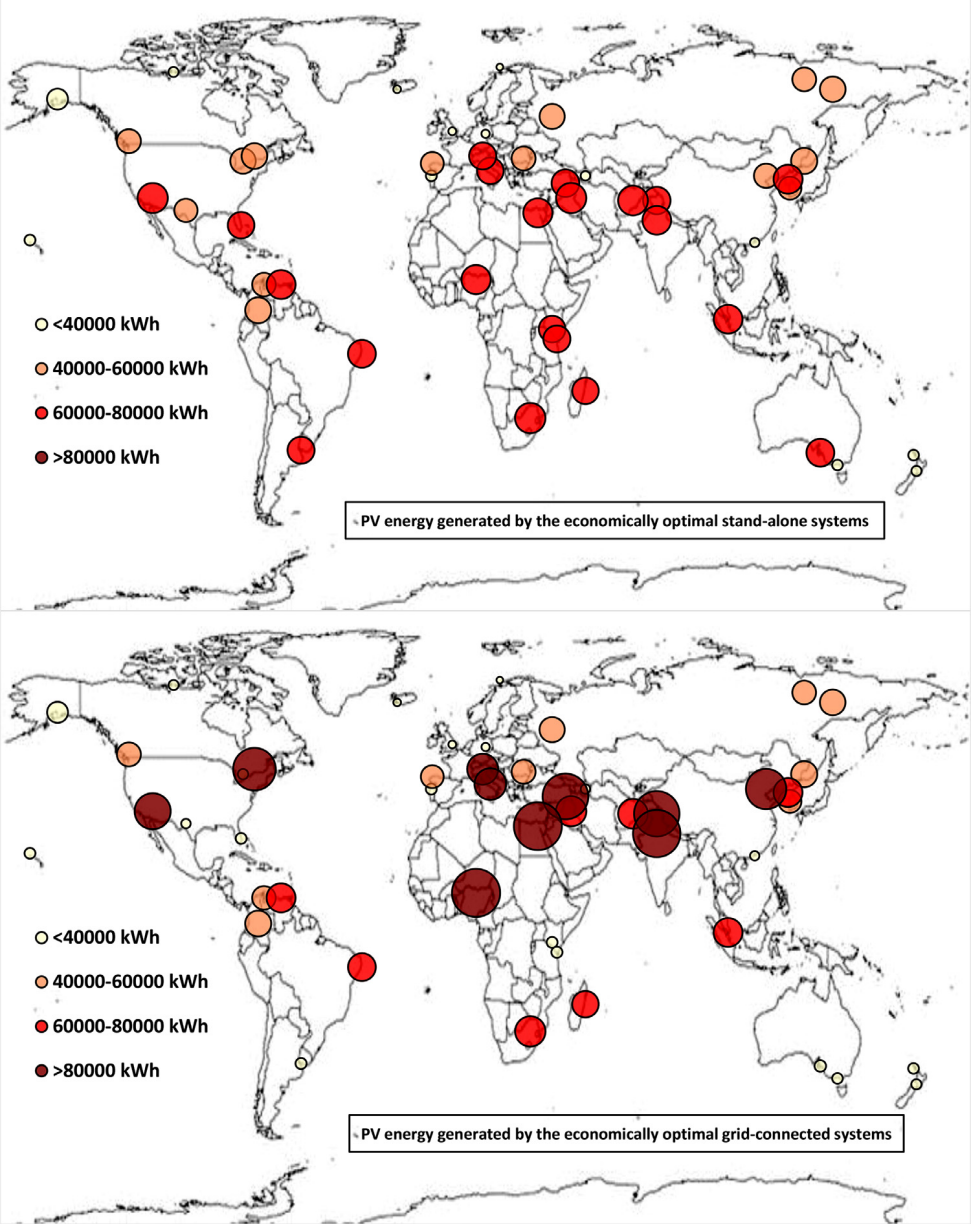
Worldwide mapping of the PV energy generated by the economically optimal stand-alone and grid-connected systems.

**Fig. 2a:**
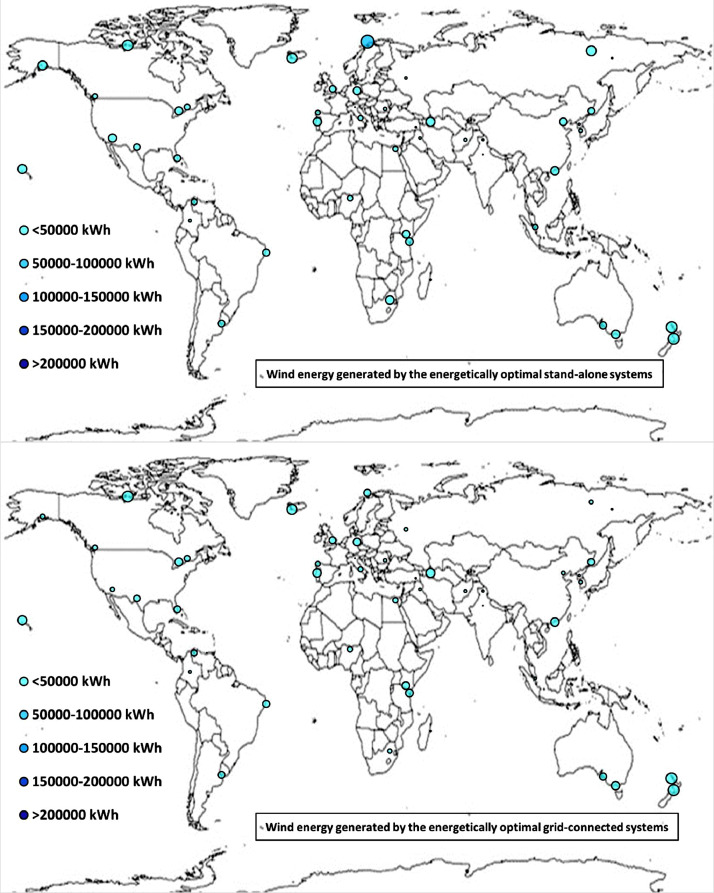
Worldwide mapping of the wind energy generated by the energetically optimal stand-alone and grid-connected systems.

**Fig. 2b:**
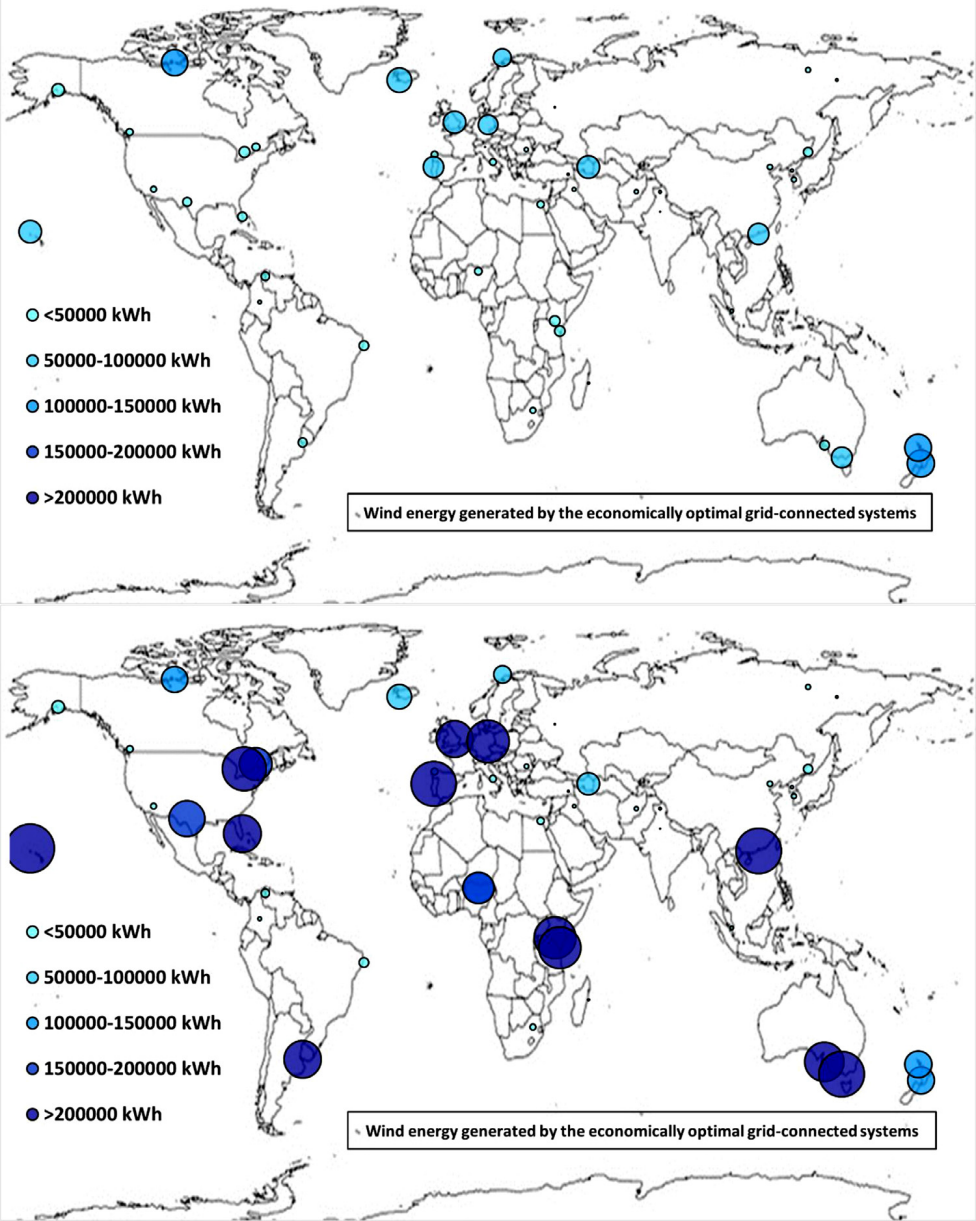
Worldwide mapping of the wind energy generated by the economically optimal stand-alone and grid-connected systems.

**Fig. 3a:**
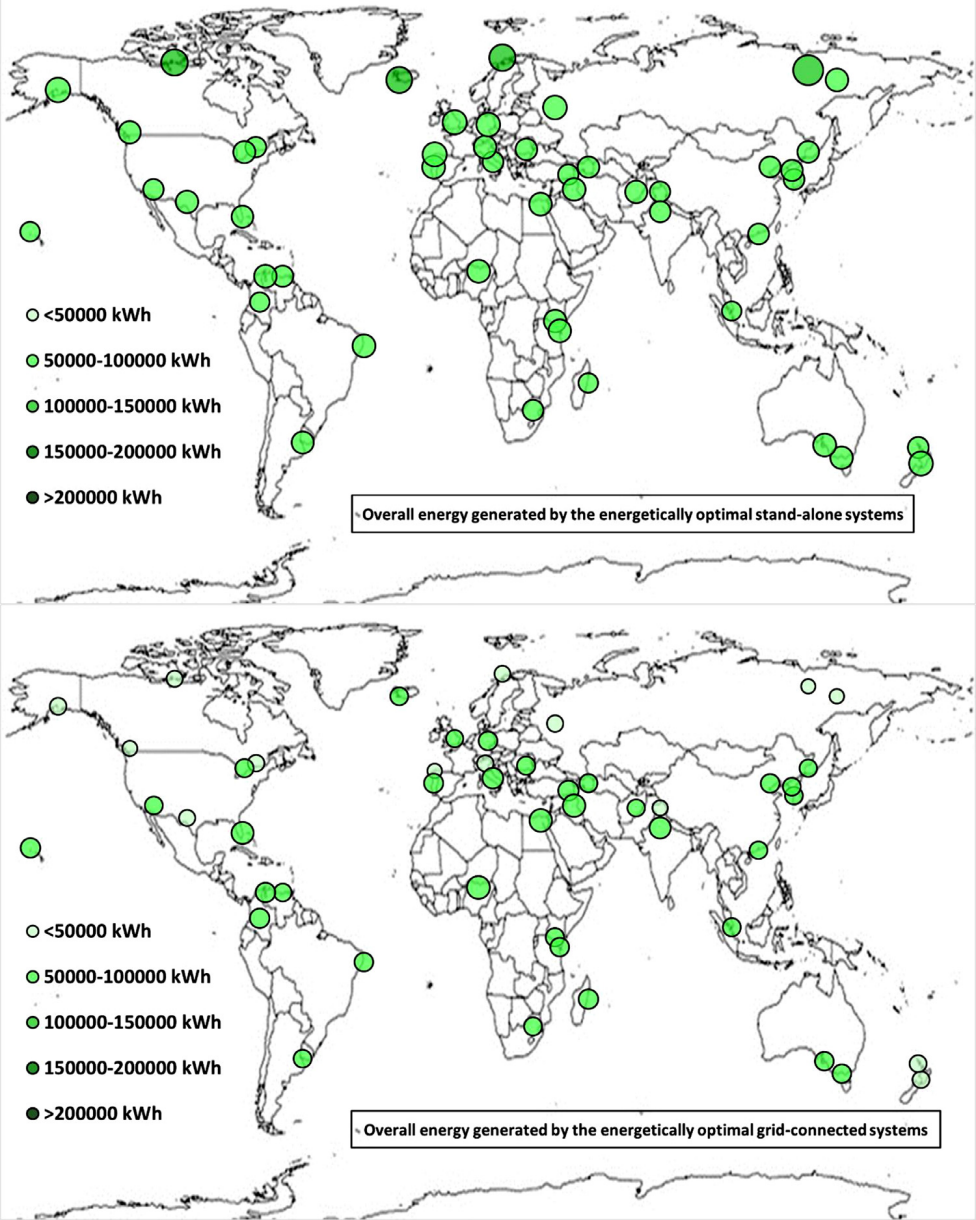
Worldwide mapping of the overall energy generated by the energetically optimal stand-alone and grid-connected systems.

**Fig. 3b:**
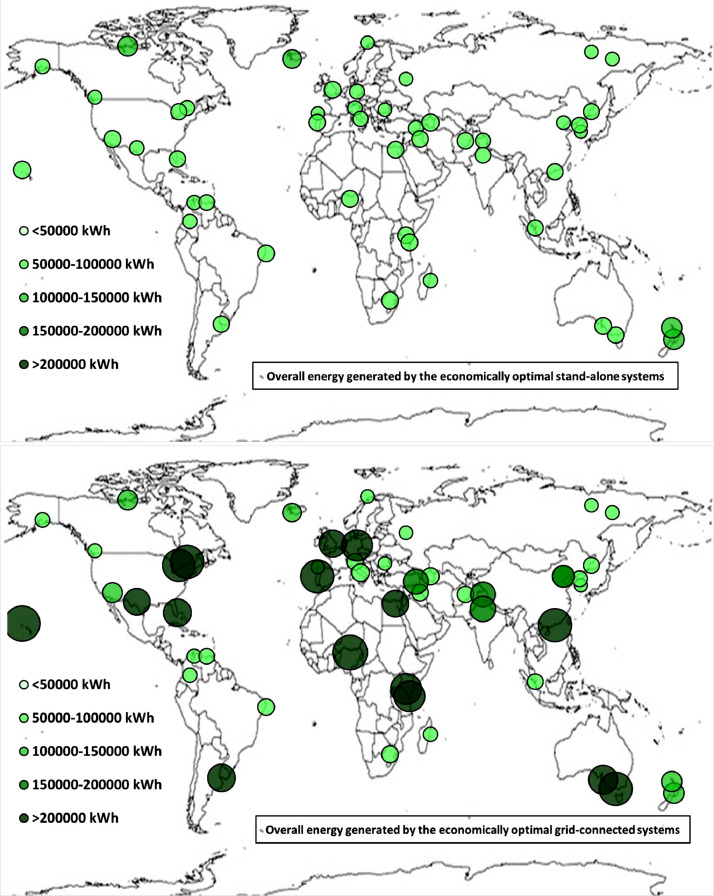
Worldwide mapping of the overall energy generated by the economically optimal stand-alone and grid-connected systems.

**Fig. 4a:**
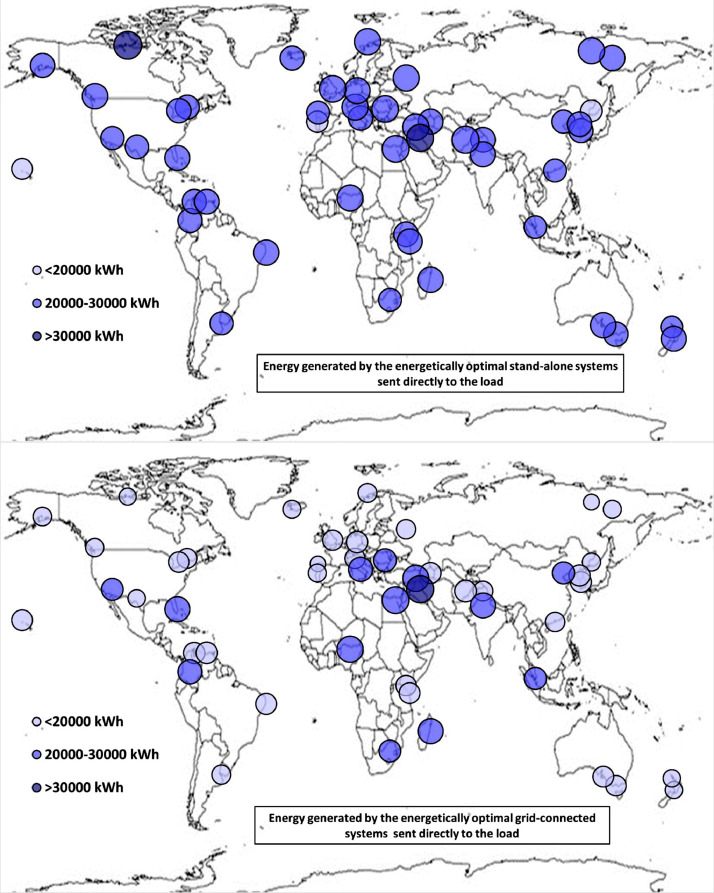
Worldwide mapping of the energy generated by the energetically optimal stand-alone and grid-connected systems sent directly to the load.

**Fig. 4b:**
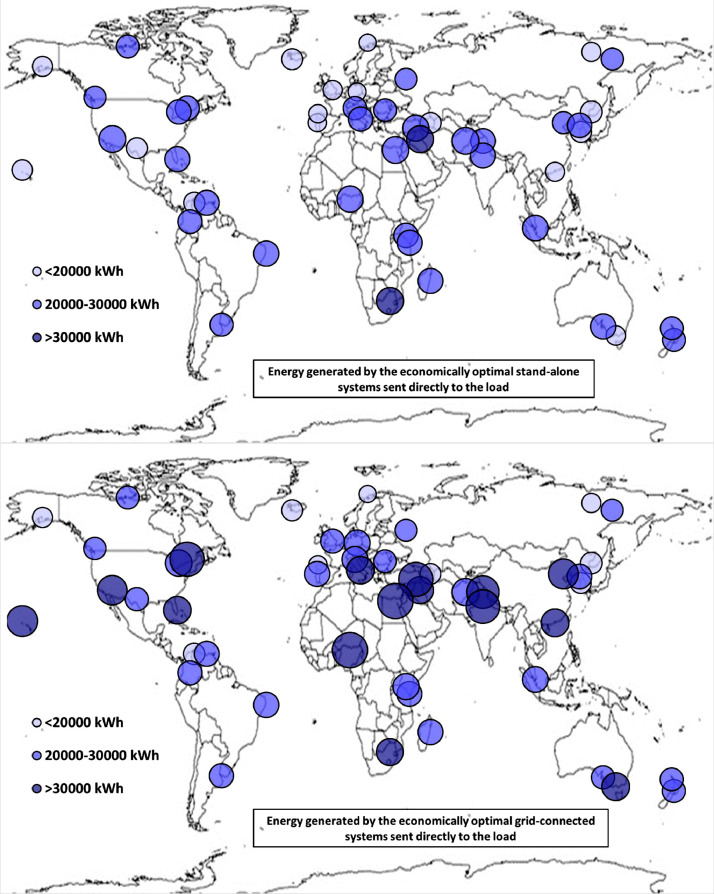
Worldwide mapping of the energy generated by the economically optimal stand-alone and grid-connected systems sent directly to the load.

**Fig. 5a:**
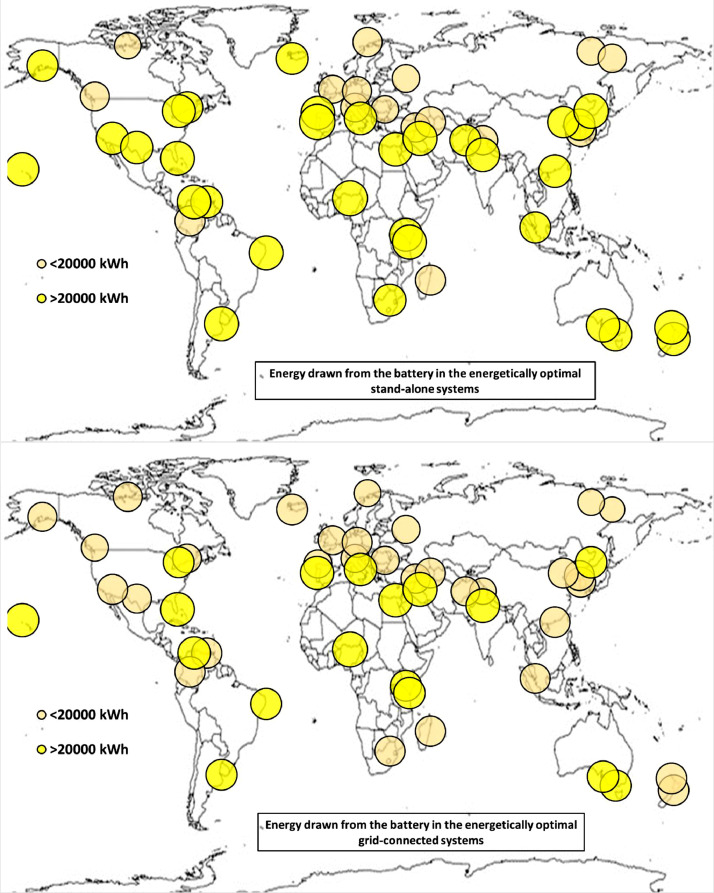
Worldwide mapping of the energy drawn from the battery in the energetically optimal stand-alone and grid-connected systems.

**Fig. 5b:**
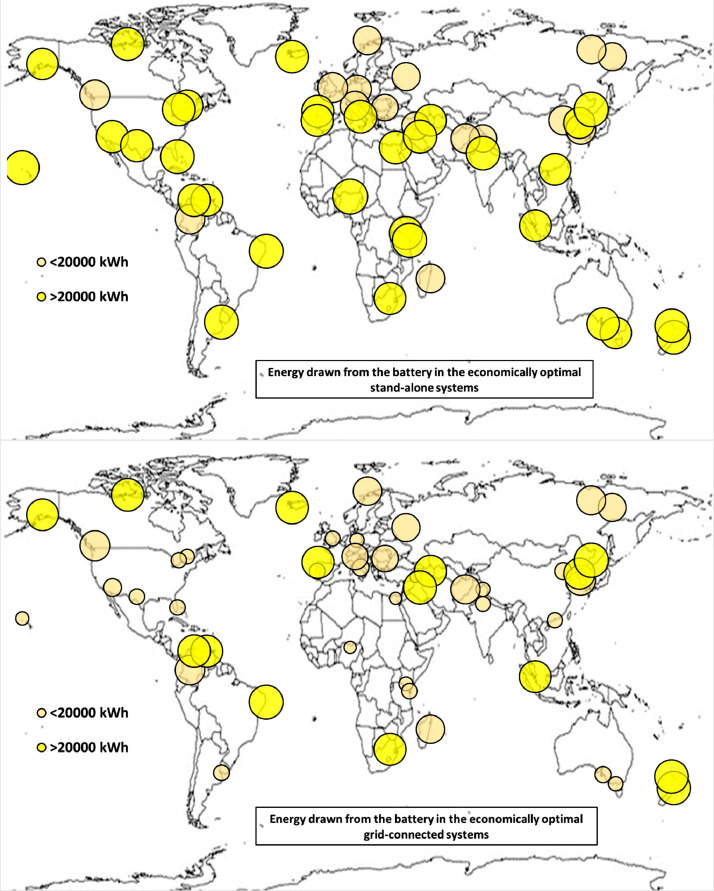
Worldwide mapping of the energy drawn from the battery in the economically optimal stand-alone and grid-connected systems.

**Fig. 6a:**
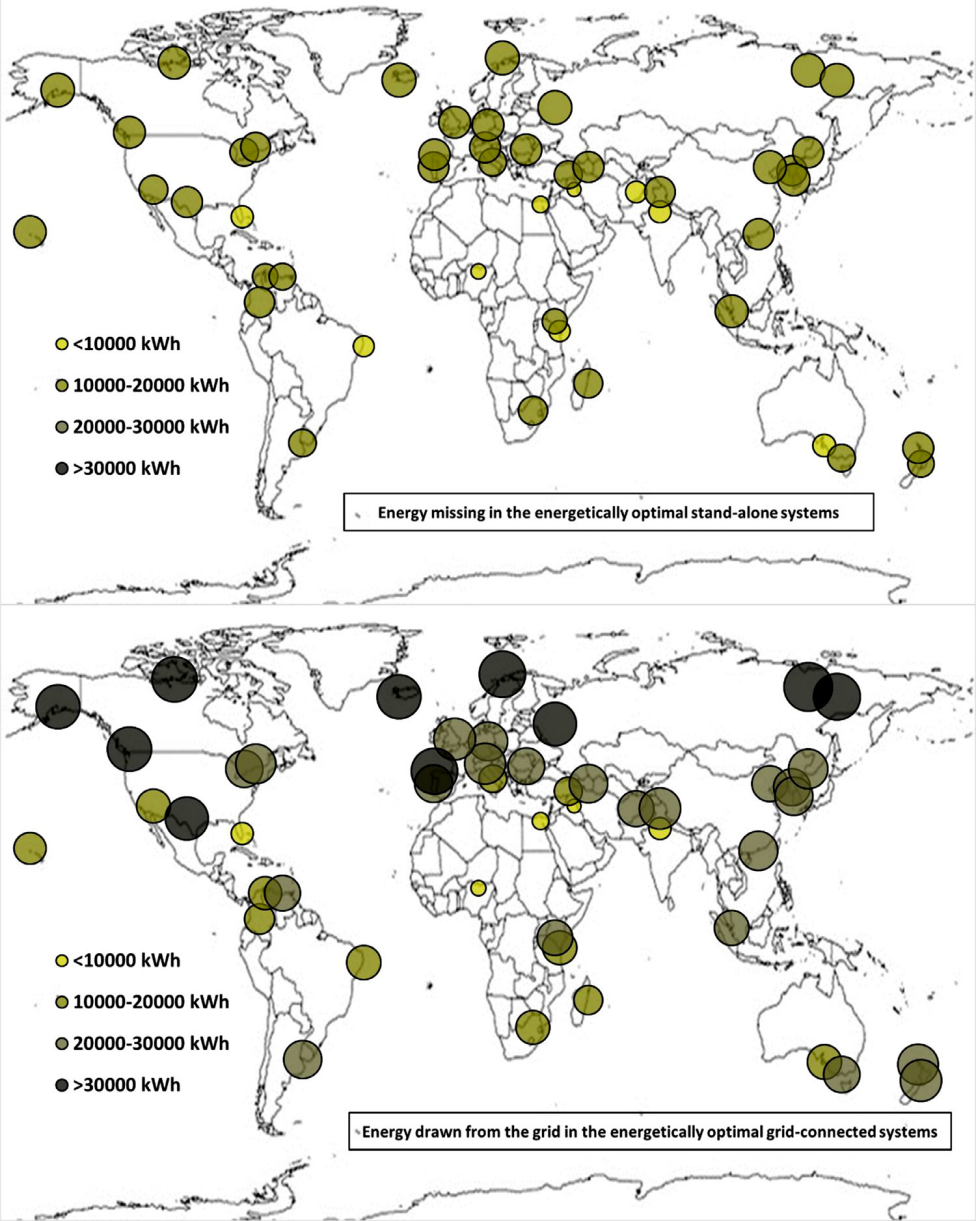
Worldwide mapping of the energy missing in the energetically optimal stand-alone systems and drawn from the grid in the energetically optimal grid-connected systems.

**Fig. 6b:**
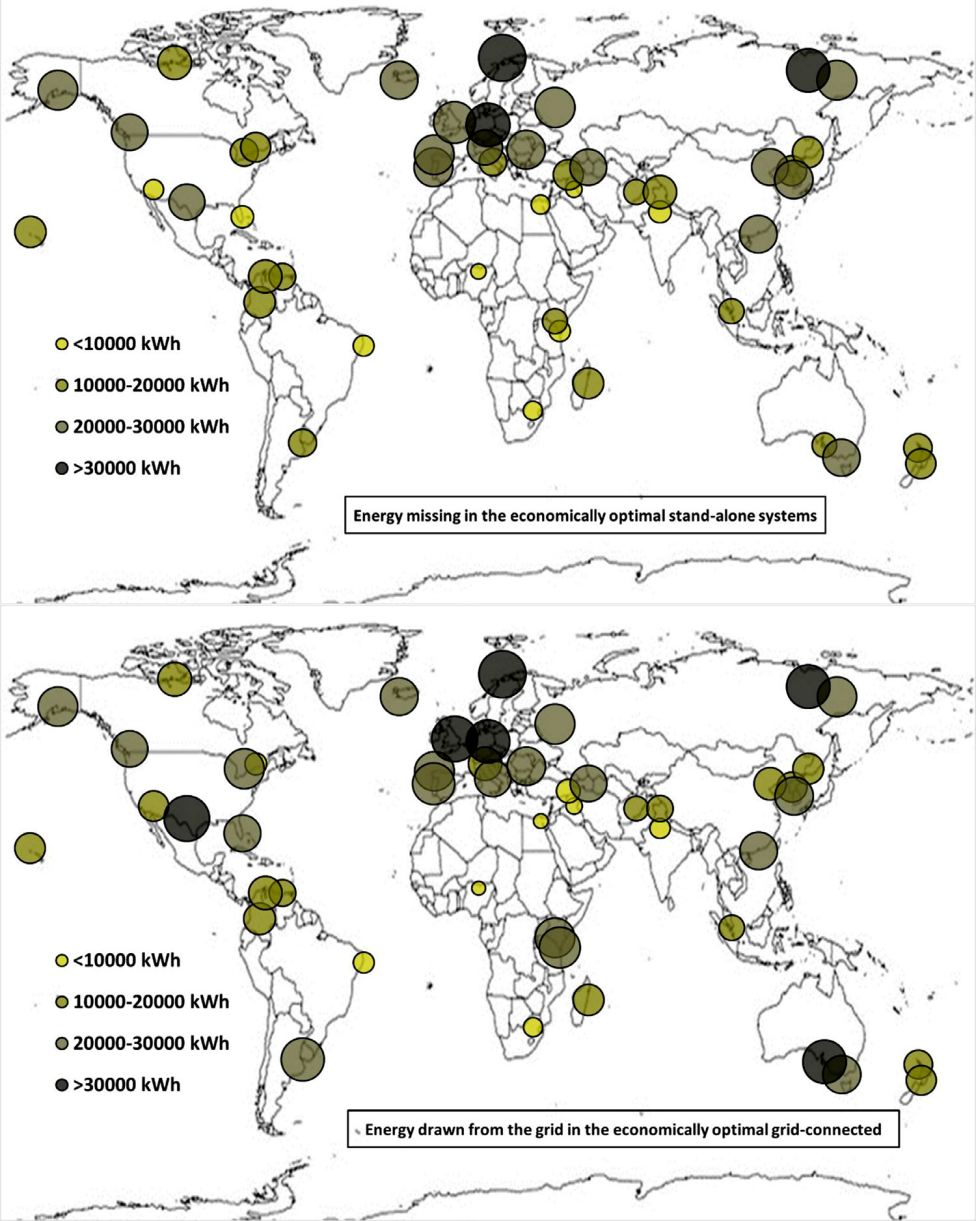
Worldwide mapping of the energy missing in the economically optimal stand-alone systems and drawn from the grid in the economically optimal grid-connected systems.

**Fig. 7a:**
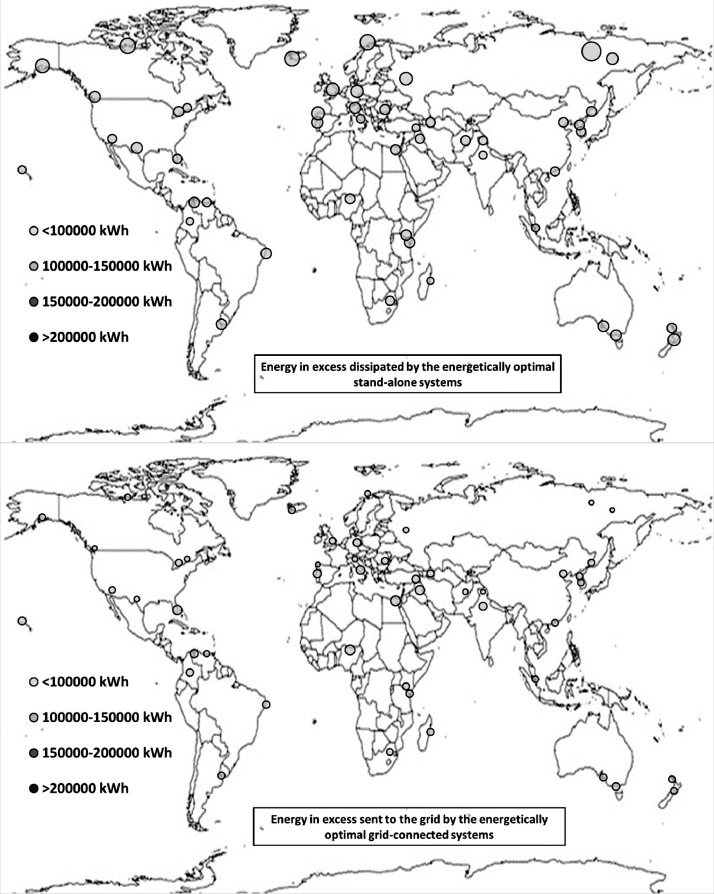
Worldwide mapping of the energy in excess dissipated by the energetically optimal stand-alone systems and sent to the grid by the energetically optimal grid-connected systems.

**Fig. 7b:**
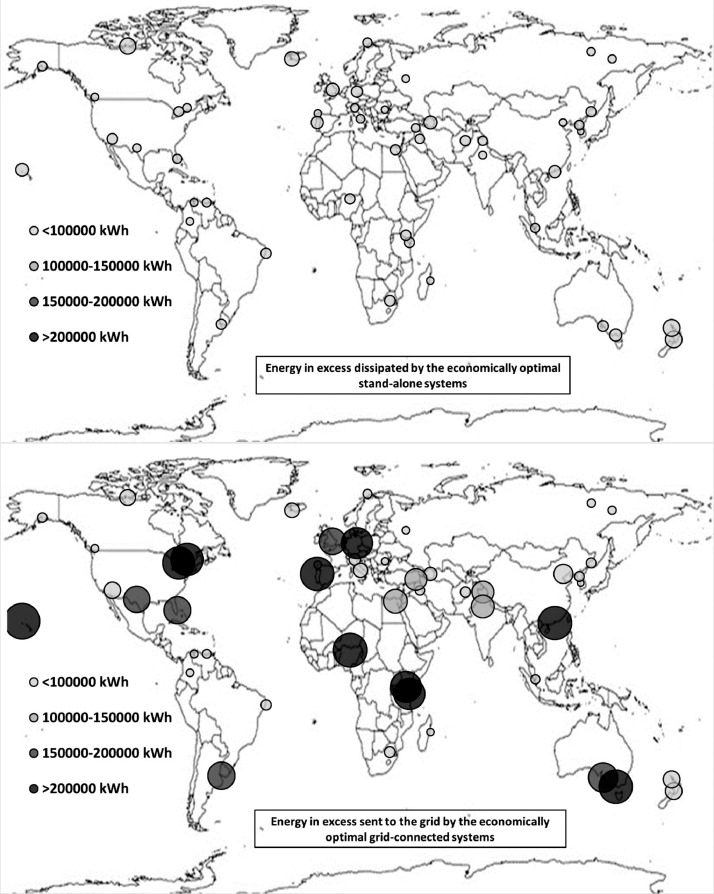
Worldwide mapping of the energy in excess dissipated by the economically optimal stand-alone systems and sent to the grid by the economically optimal grid-connected systems.

**Fig. 8a:**
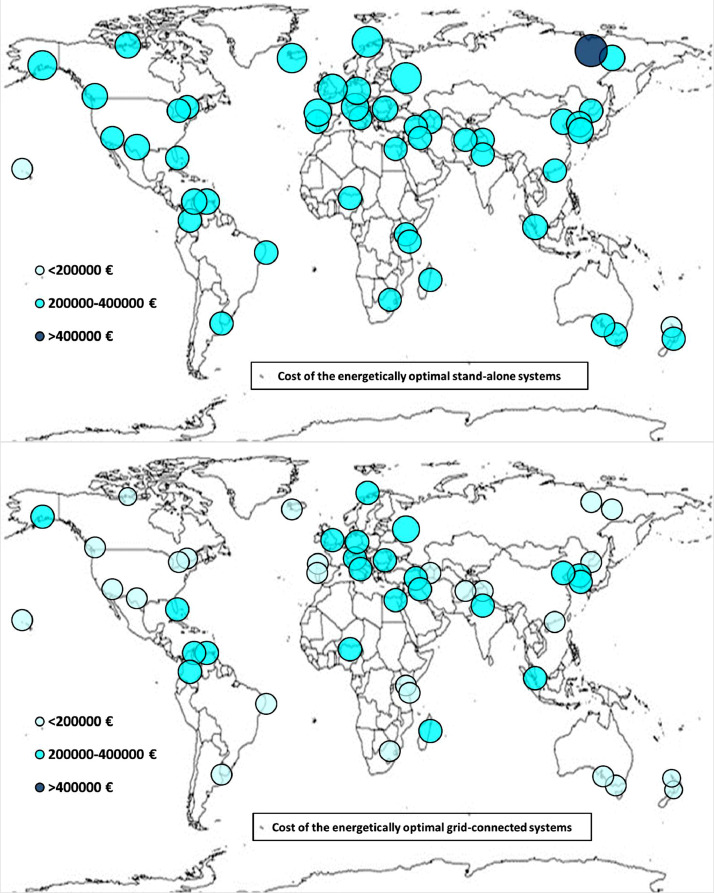
Worldwide mapping of the cost of the energetically optimal stand-alone and grid-connected systems.

**Fig. 8b:**
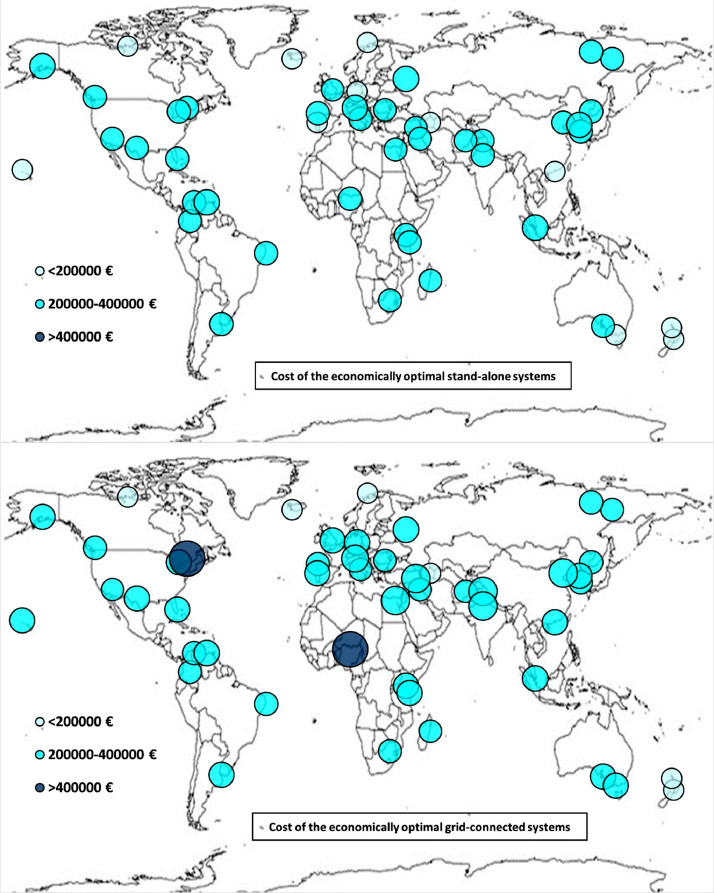
Worldwide mapping of the cost of the economically optimal stand-alone and grid-connected systems.

**Fig. 9a:**
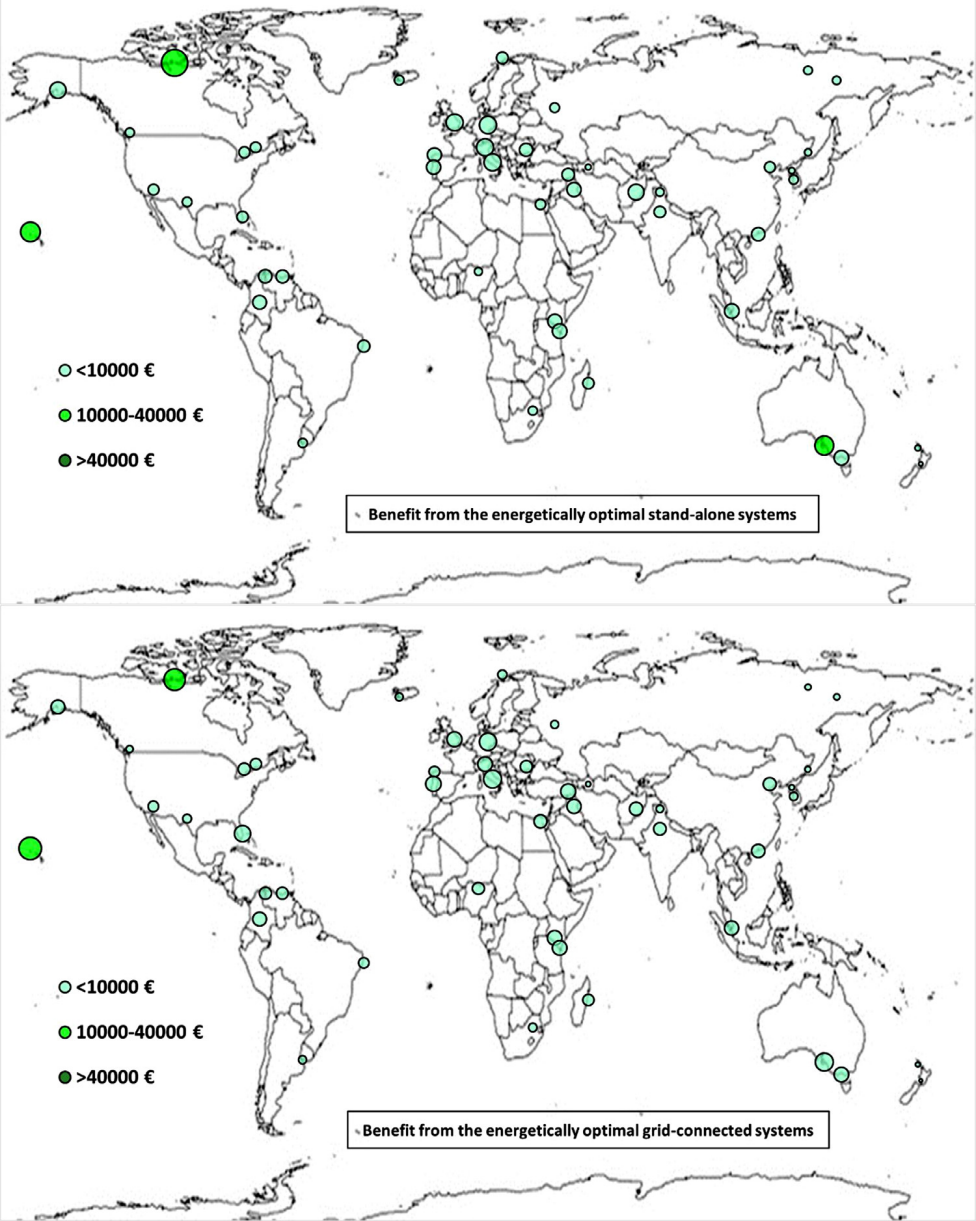
Worldwide mapping of the benefit from the energetically optimal stand-alone and grid-connected systems.

**Fig. 9b:**
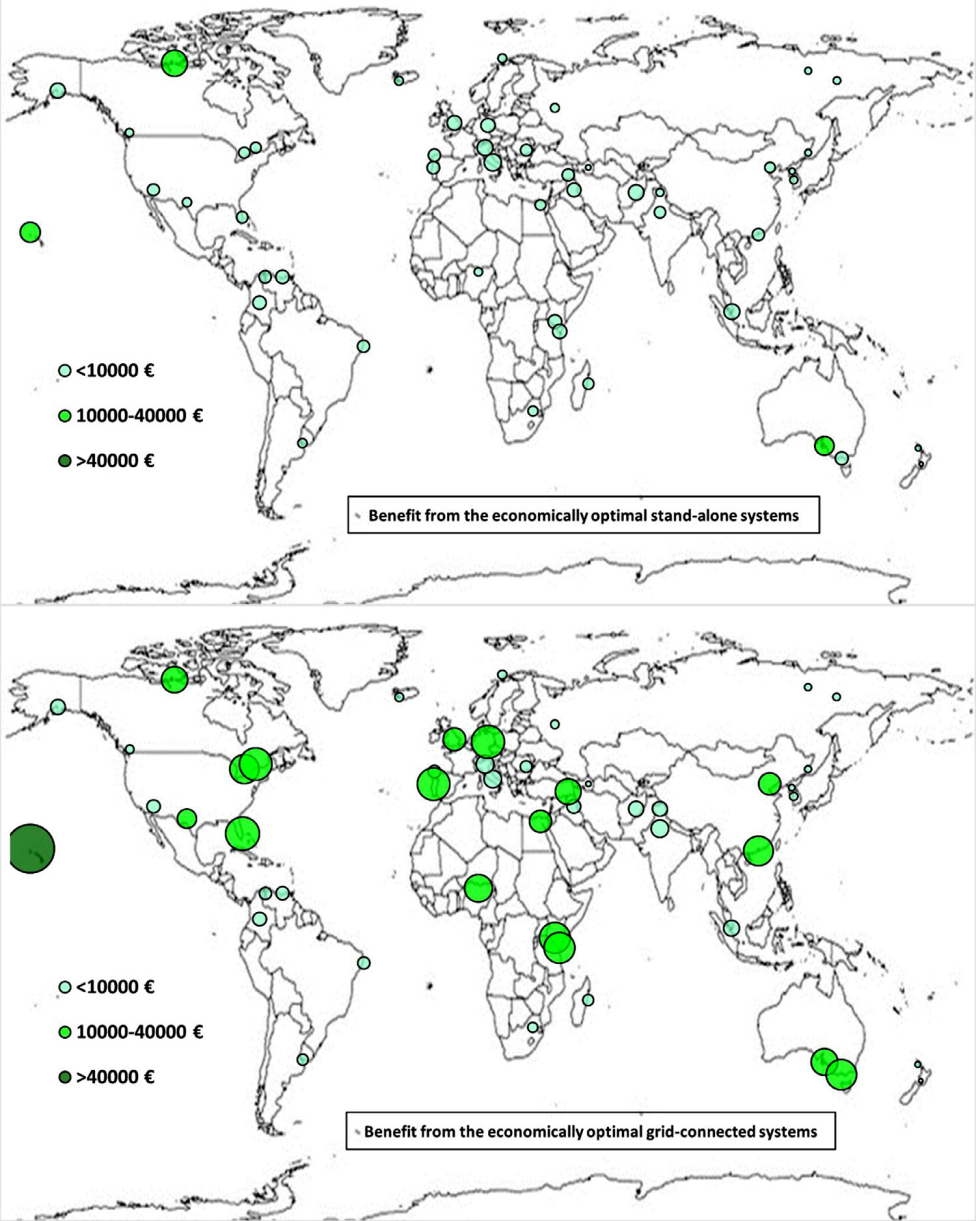
Worldwide mapping of the benefit from the economically optimal stand-alone and grid-connected systems.

The worldwide geographical maps of other energy and economic indicators are contained in the main paper [1]. The bubble size is proportional to the value of the parameter or indicator in the locality considered. For a specific parameter or indicator, the sizes of bubbles in the four images related to the energetically and economically optimal SA and GC HRES are between the absolute maximum and minimum values observed in the four datasets.

## 2 Materials and Methods

This data article shows the results of worldwide techno-economic analyses of SA and GC PV-wind HRES designed to meet the electrical energy needs of a district composed of five identical office buildings. The load requires energy for electric lighting, office equipment and vehicle charging stations.

**Fig. 10:**
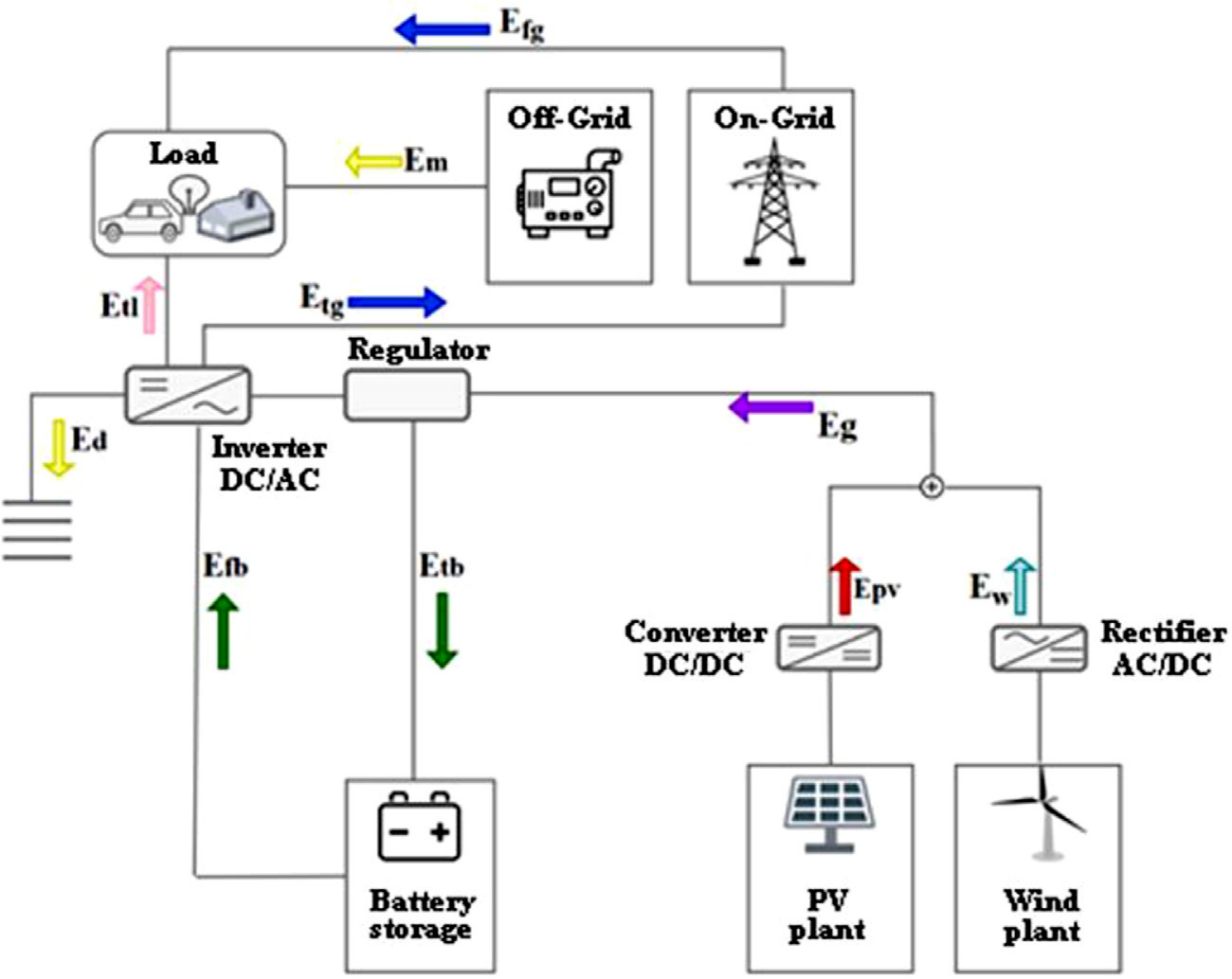
Scheme of the SA and GC PV-wind HRES with battery storage.

Fig. 10 shows a synthetic scheme of the SA and GC HRES analyzed and the relative energy flows, indicated as follows:

• in blue: the yearly energy drawn from the grid (E_fg_) and the yearly energy produced sent to the grid (E_tg_);
• in yellow: the yearly missing energy (E_m_) and the yearly energy dissipated (E_d_);
• in green: the yearly energy drawn from the battery (E_fb_) and the yearly energy sent to the battery (E_tb_);
• in pink: the yearly energy produced sent to the load (E_tl_);
• in purple: the yearly overall energy produced by the generators (E_g_);
• in red: the yearly energy produced by the PV generator (E_pv_);
• in light blue: the yearly energy produced by the wind generator (E_w_).

Once the energy produced is more than the energy needed by the load, the energy is sent to the load and the remaining part is taken to charge the battery. The energy in excess is dissipated for the stand-alone system and sent to the grid for the grid-connected system. On the other hand, when the energy generated is not able to meet the load, it is taken from the battery. The missing energy is supplied by alternative systems, using a diesel generator for the stand-alone system, or by withdrawing energy from the grid for the grid-connected system [4,5].

TRNSYS software [6] was used to simulate the system, as explained in detail in [1].

The electrical-thermal performance of the PV plant is implemented with the Type 94a, the wind plant with the Type 90 and the lithium-ion battery with the Type 47. Type 48 models the regulator and inverter, Type 15 imports the .tm2 climatic data from TRNSYS weather library and Type 25 plots the results.

A total of 343 HRES power configurations, in terms of different PV, wind and battery powers, were dynamically simulated for 48 localities worldwide. For the 16464 simulations, hourly powers from each component were summarized in yearly energies, benefits and costs, and energy and economic indicators. An energy optimization problem was formulated to find, in each locality, the most reliable system power configuration for the SA HRES. Another energy optimization problem was formulated to find, in each locality, the system power configuration with the minimum level of energy exchange with the grid for the GC HRES. Similarly, two economic optimization problems were formulated, respectively, for the SA and GC HRES, to select the system power configuration with the highest benefit-cost ratio.

## Ethics Statement

The authors gave their consent to apply the instrument and provide the necessary data.

## Declaration of Competing Interest

The authors declare that they have no known competing financial interests or personal relationships which have, or could be perceived to have, influenced the work reported in this article.

## Appendix Supplementary materials

Image, application 1
