# Supplementary material for: Energy and economic dataset of the worldwide optimal photovoltaic-wind hybrid renewable energy systems
Source: Data Brief. 2020 Nov 1;33:106476. doi: 10.1016/j.dib.2020.106476 (PMC7666328; doi:10.1016/j.dib.2020.106476)
Supplement: Supplementary file 1 [file mmc1.zip › mmc1/02 Supplementary material - Histograms.docx]

**Supplementary material – Histograms**

In the following supplementary material, additional graphical elaborations related to the worldwide energetically and economically optimal SA and GC HRES are presented. The explanations of how figures were obtained and their description and usefulness are contained in the main paper.

**Figure S.1.** PV and wind power fractions for the energetically optimal stand-alone and grid-connected systems.

**Figure S.2.** PV and wind power fractions for the economically optimal stand-alone and grid-connected systems.

**Figure S.3.** Battery power fractions and load power fractions for the energetically optimal stand-alone and grid-connected systems.

**Figure S.4.** Battery power fractions and load power fractions for the economically optimal stand-alone and grid-connected systems.

**Figure S.5.** Wind and PV energy generated by the energetically optimal stand-alone and grid-connected systems.

**Figure S.6.** Wind and PV energy generated by the economically optimal stand-alone and grid-connected systems.

**Figure S.7.** PV and wind fractions of the overall energy generated in the energetically optimal stand-alone and grid-connected systems.

**Figure S.8.** PV and wind fractions of the overall energy generated in the economically optimal stand-alone and grid-connected systems.

**Figure S.9.** PV and wind fractions of the overall energy generated in order of yearly average wind speed strength (decreasing from left to right) for the energetically optimal stand-alone and grid-connected systems.

**Figure S.10.** PV and wind fractions of the overall energy generated in order of yearly average horizontal solar radiation strength (decreasing from left to right) for the energetically optimal stand-alone and grid-connected systems.

**Figure S.11.** PV and wind fractions of the overall energy generated in order of yearly average wind speed strength (decreasing from left to right) for the economically optimal stand-alone and grid-connected systems.

**Figure S.12.** PV and wind fractions of the overall energy generated in order of yearly average horizontal solar radiation strength (decreasing from left to right) for the economically optimal stand-alone and grid-connected systems.

**Figure S.1.** PV and wind power fractions for the energetically optimal stand-alone and grid-connected systems.

**Figure S.2.** PV and wind power fractions for the economically optimal stand-alone and grid-connected systems.

**Figure S.3.** Battery power fractions and load power fractions for the energetically optimal stand-alone and grid-connected systems.

**Figure S.4.** Battery power fractions and load power fractions for the economically optimal stand-alone and grid-connected systems.

**Figure S.5.** Wind and PV energy generated by the energetically optimal stand-alone and grid-connected systems.

**Figure S.6.** Wind and PV energy generated by the economically optimal stand-alone and grid-connected systems.

**Figure S.7.** PV and wind fractions of the overall energy generated in the energetically optimal stand-alone and grid-connected systems.

**Figure S.8.** PV and wind fractions of the overall energy generated in the economically optimal stand-alone and grid-connected systems.

**Figure S.9.** PV and wind fractions of the overall energy generated in order of yearly average wind speed strength (decreasing from left to right) for the energetically optimal stand-alone and grid-connected systems.

**Figure S.10.** PV and wind fractions of the overall energy generated in order of yearly average horizontal solar radiation strength (decreasing from left to right) for the energetically optimal stand-alone and grid-connected systems.

**Figure S.11.** PV and wind fractions of the overall energy generated in order of yearly average wind speed strength (decreasing from left to right) for the economically optimal stand-alone and grid-connected systems.

**Figure S.12.** PV and wind fractions of the overall energy generated in order of yearly average horizontal solar radiation strength (decreasing from left to right) for the economically optimal stand-alone and grid-connected systems.
